# Coronary artery aneurysm complicated by pneumothorax with a history of Kawasaki disease

**DOI:** 10.1093/jscr/rjab053

**Published:** 2021-03-29

**Authors:** Shinichi Ishida, Masato Mutsuga, Takashi Fujita, Masao Ito, Sawako Okamoto, Kei Yagami

**Affiliations:** Department of Cardiac Surgery, Gifu Prefectural Tajimi Hospital, Tajimi, Gifu, Japan; Department of Cardiac Surgery, Nagoya University Graduate School of Medicine, Nagoya, Aichi, Japan; Department of Cardiac Surgery, Gifu Prefectural Tajimi Hospital, Tajimi, Gifu, Japan; Department of Thoracic Surgery, Gifu Prefectural Tajimi Hospital, Tajimi, Gifu, Japan; Department of Thoracic Surgery, Gifu Prefectural Tajimi Hospital, Tajimi, Gifu, Japan; Department of Cardiac Surgery, Gifu Prefectural Tajimi Hospital, Tajimi, Gifu, Japan

## Abstract

Kawasaki disease (KD) is a common vasculitis disorder of childhood. It can sometimes complicate coronary artery aneurysms, and treatment is required depending on the condition of stenosis. A 20-year-old man was referred for surgery with a coronary artery aneurysm and stenosis in the left coronary artery as sequelae of KD. He had a surgical history of left pneumothorax and bullae remaining on the right lung. We simultaneously performed off-pump coronary artery bypass for coronary artery stenosis and bullectomy. Coronary artery aneurysms with KD complicated by pneumothorax are rare, and we treated them using one-stage surgery.

## INTRODUCTION

Kawasaki disease (KD) is a vasculitis disease of unknown cause that mainly affects children. It causes coronary artery aneurysms and sometimes involves severe stenosis or obstruction [1]. On the other hand, spontaneous pneumothorax is a common disease particularly in young male patients. It occasionally recurs as a result of the presence of bulla [2]. Here, we report coronary artery stenosis as the sequela of KD complicated by spontaneous pneumothorax and bullae. Moreover, we performed coronary artery bypass grafting (CABG) and bullectomy in a single surgery to treat both conditions simultaneously.

## CASE REPORT

**Figure 1:**
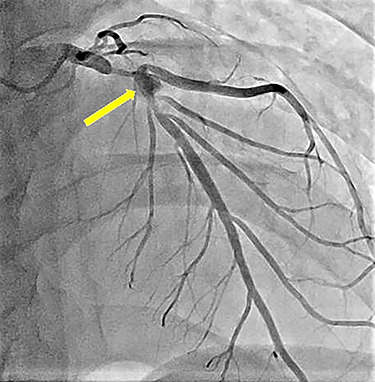
Preoperative left coronary angiography showing the coronary artery aneurysm in the left anterior descending artery (arrow) and severe stenosis at its distal and proximal sides.

**Figure 2:**
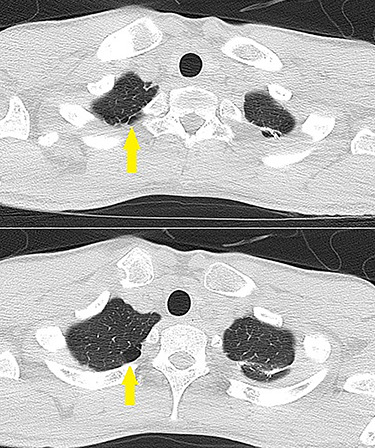
Lung CT showing bullae on the pulmonary apex of the left lung (arrows).

A 20-year-old man with a history of KD was diagnosed with a coronary artery aneurysm in his left anterior descending artery. His condition was controlled by antiplatelet and anticoagulant therapy, and he had no symptoms. However, coronary angiography showed progression of stenosis (Fig. 1), and his resting full-cycle ratio was 0.39; thus, CABG was planned. In addition, he had a history of two surgical procedures for left spontaneous pneumothorax. The first occurrence of the pneumothorax was a year ago, with recurrence within 6 months. The patient was tall and thin. Bullae also existed on his right lung (Fig. 2), which was possibly the cause of the new pneumothorax, because he had repeated pneumothorax in the other lung. Therefore, we planned bullectomy at the same time.

First, we performed CABG through median sternotomy using arterial conduits (left internal mammary artery–left anterior descending, right internal mammary artery–first diagonal branch) without cardiopulmonary bypass on the beating heart. Second, we incised the right mediastinal pleura and secured a view of the bullae using the sternal retractor when harvesting the right internal mammary artery. Using a bronchial blocker, we created temporary lung ventilation. An autosuture device was used for the resection of bullae. The postoperative course was uneventful.

## DISCUSSION

KD is a vasculitis that occurs in childhood, and coronary artery aneurysms are known as an important complication. Meanwhile, spontaneous pneumothorax is a common disease in young males. In the present case, there was a significant coronary artery stenosis that needed treatment. By contrast, although there was no right pneumothorax, the left side relapsed, and it is possible that the right side may also get affected in the future. Therefore, after consulting with the patient, we decided to perform prophylactic bullectomy to prevent bleb rupture and spontaneous pneumothorax. We detected two important clinical issues from the case reported here.

First, coronary artery aneurysm as a sequela of KD is complicated by spontaneous pneumothorax. KD with coronary artery aneurysms is a relatively rare disease; however, pneumothorax is common. High fever and systemic various symptoms are known symptoms of KD, and sequelae are generally arterial disorders such as coronary artery aneurysms rather than respiratory disease such as pneumothorax [1, 3, 4]. In this case, a pneumothorax occurred after more than a decade after the treatment for KD. In addition, his body shape was typical for spontaneous pneumothorax. Therefore, it is more likely that the two diseases were complicated accidentally rather than being related. However, because there have been no reports of cases of pneumothorax as sequelae of KD, we will be careful to observe whether there are any similar cases in the future.

Second, coronary artery stenosis and bullae were treated at the same time by performing off-pump CABG and bullectomy using one-stage surgery. These two surgeries are not very rare procedures, but because there are no existing reports on performing them simultaneously, consideration as to how to approach and secure the operational field was necessary. With regard to the approach, a median sternotomy or anterolateral thoracotomy was considered. Because we had to harvest the bilateral internal mammary artery, create an anastomosis to the left anterior descending and diagonal branch and resect the bullae on the right lung, we choose the median sternotomy approach. If the bullae had been on the left lung, a left anterolateral thoracotomy (e.g. minimally invasive direct CABG) would have been an option. To secure the operational field, we used one-lung ventilation and a sternal retractor for the right internal mammary artery. Because the bullae were on the pulmonary apex, they were easy to visualize. We considered adding ports to the autosuture device in case the bullae had been on the side of the lung. In addition, if the procedure had been more complex or the patient had poor breathing function, cardiopulmonary bypass or extracorporeal membrane oxygenation would have been required [5].

In conclusion, we encountered a patient with coronary artery aneurysms resulting from KD and bullae as the possible of the cause of pneumothorax. Both were therapeutic indications. We performed CABG and bullectomy as a one-stage surgery to treat both diseases simultaneously, and the postoperative course was uneventful. There is a current trend for low-invasive surgery, and there are benefits to operating two organs from the same wound. Because it is possible to perform bullectomy with cardiac surgery, we should consider performing resection when a bulla is observed on computed tomography (CT).
